# Real-World Safety and Effectiveness of Elexacaftor, Tezacaftor, and Ivacaftor in People with Cystic Fibrosis and Advanced Lung Disease: A Two-Year Multicenter Cohort Study

**DOI:** 10.3390/ijms262110513

**Published:** 2025-10-29

**Authors:** Sonia Volpi, Maura Ambroni, Roberto Buzzetti, Giuseppe Cimino, Andrea Gramegna, Maria Cristina Lucanto, Pietro Ripani, Mirco Ros, Donatello Salvatore, Elena Spada, Cesare Braggion

**Affiliations:** 1 Cystic Fibrosis Centre, Azienda Ospedaliera Universitaria Integrata, Piazzale Stefani 1, 37126 Verona, Italy; 2 Cystic Fibrosis Centre, M. Bufalini Hospital, 47521 Cesena, Italy; maura.ambroni@auslromagna.it; 3 Freelance Epidemiologist, 24100 Bergamo, Italy; robuzze@gmail.com; 4 Cystic Fibrosis Centre, AOU Policlinico Umberto 1, 00155 Rome, Italy; gi.cimino@policlinicoumberto1.it; 5 Respiratory Unit and Cystic Fibrosis Center, Fondazione IRCCS Cà Granda Ospedale Maggiore Policlinico, 20122 Milano, Italy; 6 Cystic Fibrosis Center, G. Martino University Hospital, 98125 Messina, Italy; 7 Cystic Fibrosis Centre, San Liberatore Hospital, 64032 Atri, Italy; 8 Cystic Fibrosis Unit, Ca’ Foncello Hospital, 31100 Treviso, Italy; mirco.ros@aulss2.veneto.it; 9 Cystic Fibrosis Centre, San Carlo Hospital, 85100 Potenza, Italy; donatello.salvatore@libero.it; 10 Laboratorio della Conoscenza Carlo Corchia, 50132 Florence, Italy; 11 Scientific Direction, Italian Cystic Fibrosis Foundation, 37126 Verona, Italy

**Keywords:** cystic fibrosis, real-world evidence, elexacaftor-tezacaftor-ivacaftor, long-term effects

## Abstract

Elexacaftor/tezacaftor/ivacaftor (ETI) is a cystic fibrosis (CF) transmembrane conductance regulator modulator, which has shown efficacy in people with CF (pwCF) carrying the F508del (F) variant, both in homozygosity and heterozygosity with a minimal function (MF) variant. Limited data exist on the effects of ETI in pwCF with advanced lung disease. Our aim was to investigate ETI safety and effectiveness in this patient group in a real-life setting over 2 years. A multicenter observational cohort study was designed to gather real-world information on the effect of ETI treatment on CF patients (aged >12 years, genotype: F/MF mutation) with advanced lung disease as defined by a FEV1 < 40% predicted. Retrospective demographic and clinical data were recorded for the two years preceding and the two years following ETI initiation. The following outcomes were investigated: treatment-associated adverse events (AEs), drug interruptions (temporary or permanent), variations in percent predicted FEV1 (ppFEV1), sweat chloride concentration (SwCl), antibiotic use, body mass index (BMI), and quality of life. A total of 124 (51.6% males) pwCF were treated with ETI over 2 years. The median (IQR) age and ppFEV_1_ were 34 (26, 43) years and 34 (29, 41) percentage points, respectively. ETI was discontinued in two pwCF due to lung transplantation, and temporarily interrupted in two because of skin rash, and in three following elevated levels of aminotransferase. Most AEs were mild and short-lasting. In 12.1% pwCF, we registered an increase greater than twice the upper limit of the normal range in alanine aminotransferase, and in 16% we registered an increase in conjugated bilirubin with no increase in aminotransferase. Both increases were recurrent in about half of the subjects. The mean differences (95% CI) for ppFEV_1_ and SwCl, assessed as mean values in the pre-ETI and ETI treatment periods, were +11.8 (11.1 to 12.6) and −43.7 (−47.6 to −39.9) mmol/L. A modest increase in ppFEV_1_ persisted during the second year of treatment. Number of oral and IV antibiotic cycles/year, as well as hospitalizations/year, decreased significantly from 3.6 to 1.2, from 2.4 to 0.6, and from 2.1 to 0.5 during ETI treatment. A total of 8 of 16 (50%) pwCF were taken off the waiting list for lung transplantation, and significant reductions in the percentages of pwCF using long-term oxygen therapy and non-invasive ventilation were observed. A poor concordance between ppFEV1 and SwCl was found. In only 3/82 (3.7%), subjects with chronic airway infection by *Pseudomonas aeruginosa* cultures were always negative during ETI treatment. In CF patients with advanced lung disease on ETI treatment, we observed an improvement in a number of clinically significant outcomes over a 2-year study period. However, several additional observations, such as liver dysfunction, variable degrees of lung function improvement, and limited impact on chronic airway infection, underscore the fact that the benefit–risk profile of ETI treatment in cystic fibrosis patients with advanced lung disease has not been fully elucidated and warrants prolonged-term monitoring.

## 1. Introduction

Cystic fibrosis (CF) is an autosomal recessive disease caused by sequence variants involving the CF transmembrane conductance regulator (CFTR) gene, resulting in reduced function of the CFTR protein anion channel present in different epithelial linings [1,2,3,4]. Over 2000 CFTR mutations have been identified to date, with some being more common than others. Nearly 90% of CF patients carry at least one mutation in the F508del-CFTR allele [5].

In an effort to address the underlying causes of the disease, CFTR modulators have been developed and applied in clinical trials. CFTR potentiators act on gating defects associated with some CFTR mutant proteins, whereas CFTR correctors improve limitations in CFTR protein processing and trafficking associated with other mutations [6,7]. Double or triple associations of different CFTR modulators have recently been studied. Particularly, the association of elexacaftor (a CFTR corrector), tezacaftor (a CFTR corrector), and ivacaftor (a CFTR potentiator) has been tested in clinical trials in CF patients with at least one F508del-CFTR allele [8,9,10,11]. Treatment with elaxacaftor/tezacaftor/ivacaftor (ETI) has been associated with improvement in lung function, sweat chloride concentration, and respiratory symptoms in CF patients homozygous for 508del-CFTR [11]. These results were apparent during the first months of treatment, were maintained over time, and confirmed in large patient registry datasets [12,13].

However, clinical trials’ inclusion criteria usually demand baseline percent predicted FEV1 (ppFEV_1._ )in the 40–90 range, thus excluding patients with advanced lung disease. Only a few small real-life studies investigated the effects of ETI in severe CF lung disease [14,15]. Also, three French studies on CF patients with advanced lung disease and either the F508del or a non-F508del mutation showed a degree of improvement in clinical status after ETI at 3 and 12 months and 4–6 weeks of treatment, respectively [16,17,18].

The aim of this study was to assess the real-world long-term safety and effectiveness of ETI therapy in a large cohort of Italian patients with severe CF carrying F508del and a minimal function (MF) mutation by comparing the two years before and after the start of treatment.

## 2. Results

A total of 124 subjects were enrolled; none had been treated with a CFTR modulator before. All were compound heterozygous for the F508del and an MF mutation and aged 12 years or older. Main clinical and demographic data, obtained at the start of ETI treatment (T_0_), are shown in Table 1. Sixteen (12.9%) were on the lung transplant waiting list, and 38.6% were treated with long-term oxygen therapy. The median (IQR) number of either out- or in-patient visits was 8 (5, 10) before and 7 (4, 9) after the start of ETI.

### 2.1. Safety

Two patients discontinued ETI after 11 and 14 months because of lung transplantation. A temporary interruption occurred in two and three pwCF because of skin rash and elevated levels of aminotransferase, respectively. These adverse effects resolved after stopping ETI for 2–4 weeks. No deaths occurred during the study period.

Table 2 provides an overview of the adverse therapy-related events. One 49-year-old man reported anxiety and depression with suicidal ideation 9 months after the start of ETI. During the same period, he reported diffuse joint pain, particularly in the hands. Before the start of modulator therapy, he had taken an anxiolytic drug on an as-needed basis. Symptoms disappeared after an ETI dose reduction. Joint pain was reported by three more pwCF: although symptoms persisted, all patients were able to continue ETI treatment using symptomatic treatment. All other adverse events were generally mild and short.

In 99 and 81 pwCF, the values of aminotransferase and conjugated and total bilirubin were reported, respectively. The median (IQR) number of ALT and conjugated, and total bilirubin measurements were 5 (3, 8), 5 (2, 7), and 5 (3, 8), respectively. Elevated levels of alanine aminotransferase that were greater than three times the ULN occurred in 6/99 (6.1%) patients: 2/6 patients had mild liver disease and a concurrent increase in conjugated bilirubin greater than two times the ULN.

In 12/99 (12.1%) pwCF, an increase in alanine aminotransferase greater than two times the ULN was recorded. In 6/12 patients, the increase in ALT occurred at least two times during the therapy with ETI. When levels of conjugated bilirubin were reported, in 13/81 (16.0%) patients, at least one increase greater than two times the ULN was recorded without any increase in aminotransferase or y-glutamyl transferase. In 7/13 patients, the isolated increase in conjugated bilirubin occurred from two to five times during ETI treatment. In four and 1/13 patients, the medical charts reported a mild or moderate liver disease, respectively. No patient with an increase in conjugated bilirubin interrupted or discontinued the CFTR modulator. A total of 7/49 (14.3%) pwCF had at least one increase in creatin kinase greater than two times the ULN.

### 2.2. Effectiveness

Table 3 reports the row means of the patients’ averages during the pre-ETI and ETI treatment periods, and the estimated mean differences between periods (ETI vs. pre-ETI). The ppFEV_1_ increased by 11.8 percentage points (95% CI: 11.1 to 12.6) over the two-year period after modulator initiation, and ppFVC increased by 13.3 percentage points (95% CI: 12.4 to 14.1). In contrast, sweat chloride concentration decreased by an average of 43.7 mmol/L (95% CI, −47.6 to −39.9), as did the use of oral and intravenous antibiotics. The number of hospitalizations was reduced on average by 1.4 per year, resulting in approximately 20 fewer hospital days annually. The mean differences (95% IC) in oral and iv antibiotics were −26.7 (−32.4 to −21.0) and −23.7 (−27.6 to −19.9) days/year, respectively.

Supplementary Figure S1 shows the individual mean differences in ppFEV_1_. The mean differences in ppFVC and FEV_1_/FVC were coherently with the improvement in ppFEV_1_. The SpO_2_/FiO_2_ ratio increased significantly after initiating ETI treatment. The mean variations (95% CI) in CFQ-R RD and BMI z-score were +23.2 (20.4 to 26.2) and 0.65 (0.61 to 0.69), respectively.

Supplementary Figure S2 plots the cumulative percentage of subjects who increased their ppFEV_1_ by at least 5 or 10 percentage points. The figure shows that at minimum 50% of subjects obtained an increase of 5 or 10 ppFEV_1_ units after 13 and 26 weeks of treatment, respectively. After a rapid increase, the cumulative percentage of pwCF who achieved these thresholds kept slowly increasing until a plateau was reached.

**Figure 1:**
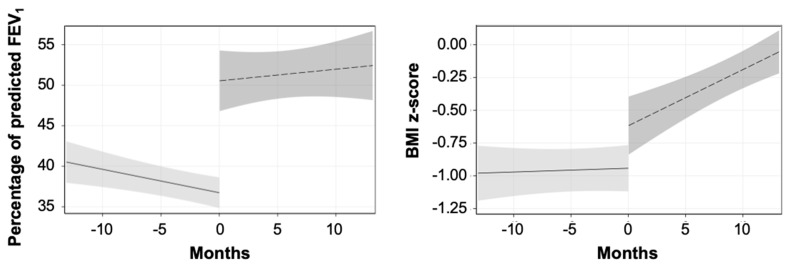
Interrupted time series (ITS) analysis: percentage of predicted FEV_1_ (**left**) and BMI z-score (**right**) with 95% CI (gray areas) by time (months) before and after beginning of therapy.

Figure 1 shows the results of the ITS analysis. The values of ppFEV_1_ decreased before and increased after the start of ETI, whereas the BMI z-score was approximately stable (about −1.0) before and rapidly increased after the start of therapy. Considering the period between 6 months before and after the beginning of ETI therapy, the mean (95% CI) increases in ppFEV_1_ and BMI z-score were of 12.9 (11.0 to 14.8) percentage points and 0.60 (0.51 to 0.69) standard deviations, respectively.

On average, the effect of ETI therapy on ppFEV_1_ and BMI was rapid during the first six months and then tended to stabilize. Between treatment initiation (T0) and six months (T1), the mean increase in ppFEV_1_ was 16.8 percentage points (95% CI: 14.4 to 19.2) and the mean increase in BMI z-score was 0.73 (95% CI: 0.59 to 0.86). In contrast, between six months and approximately two years of treatment (T1–T2), the mean increases were smaller: 2.3 (95% CI: 0.87 to 3.8) and 0.05 (95% CI: −0.05 to 0.14), respectively.

Supplementary Figure S3 shows the relationship between the mean differences in ppFEV_1_ and SwCl between the mean values of the pre-ETI and ETI treatment periods. The figure shows an increase equal to or greater than five percentage points of ppFEV_1_ and a decrease greater than 20 mmol/L of SwCl (red lines). Fifteen subjects had an increase in ppFEV_1_ of less than five percentage points, but 9/15 had a decrease in SwCl greater than 20 mmol/L. Most of the pwCF had an increase in ppFEV_1_ equal to or greater than five percentage points, but 11/79 had a decrease in SwCl less than 20 mmol/L. The concordance index (Cohen’s K) was 0.247.

Coherently with the substantial improvement in lung function and reduction in antibiotic therapy, 8 of 16 (50%) pwCF were taken off the waiting list for lung transplantation. Moreover, by the end of follow-up, the proportion of pwCF requiring long-term oxygen therapy and non-invasive ventilation had decreased from 38.6 to 16.7% and from 13.9 to 5.2%, respectively (Table 4). As reported in Table 4, the use of daily chronic medications decreased significantly during ETI treatment.

We considered 770 and 592 sputum or pharyngeal swabs as cultures for the pre-ETI and ETI treatment periods, respectively. Of these, 522/770 (68%) and 342/592 (58%) cultures were positive for PA. Among 82 pwCF with at least three cultures in each 2-year period, 59 (72%) and 53 (65%) pwCF had more than 50% of their cultures come back as positive for PA before and during ETI treatment, respectively. In only 3/82 (3.7%) subjects, cultures were always negative during ETI treatment.

## 3. Discussion

Over 24 months, treatment with a triple CFTR modulator combination was associated with a low rate of therapy interruptions or discontinuations, with the majority of reported adverse events being mild and short-lasting. But recurrent changes in liver biochemistry raised concern about worsening liver disease. In our large cohort of pwCF with advanced lung disease, we observed significant and sustained improvements in lung function, antibiotic therapy requirements, nutritional status, and daily respiratory symptoms. This improvement was sufficiently clinically relevant to allow about 50% of pwCF to be taken off a lung transplantation waiting list, or to stop long-term oxygen therapy and non-invasive ventilation.

### 3.1. Safety

The adverse events reported in patient charts by the CF center physicians were mild and short-lived in most cases, and they were consistent with the common manifestations and complications of cystic fibrosis. In contrast to other studies on pwCF with advanced lung disease, persistent joint pain was reported in four patients in our study [14,15,18,19,20]. This symptom was often coexistent with myalgia. The prevalence and pathogenesis of arthropathy in cystic fibrosis remain unclear and are probably under-reported [21]. Given the clinical benefits associated with ETI treatment, patients with joint pain continued modulator therapy by managing symptoms with analgesics. This was also true for a 49-year-old man who experienced anxiety and depression with suicidal ideation: after a dose reduction, he was able to continue ETI treatment. His pre-existing anxiety disorders did not suggest a causal link with the CFTR modulator [22].

Liver enzymes were monitored during the first and second year of ETI treatment in at least 65% of the pwCF in our study. In addition to a significant increase in aminotransferase levels, comparable to that reported in other studies on pwCF with advanced lung disease, we observed isolated increases in conjugated bilirubin [14,15,18,19,20]. These increases occurred in 13/81 (16.0%) pwCF at least once, and they were greater than twice the ULN. These abnormalities were recorded in the absence of a concurrent increase in aminotransferase or y-glutamyl transferase. In 7/13 patients, the increase in conjugated bilirubin recurred during ETI treatment. Only one patient exhibited an increase in unconjugated bilirubin, but evaluation for Gilbert’s syndrome was not considered during the study period [23].

Increases in liver enzymes and bilirubin are well known side-effects of ETI therapy and are only temporary in most cases [14,15,18,19,20]. We did not monitor changes in aminotransferase and bilirubin before the start of ETI, and so these increases could only be presumed to be related to the modulator treatment. A causative role for antibiotic therapy is unlikely, given the need for antibiotics, and the number of antibiotic days significantly decreased after the initiation of ETI. Two studies reported mild rises in median ALT, AST, and total and conjugated bilirubin, sustained over one year of ETI therapy, whereas increases greater than twice the ULN were few [24,25]. In homozygous patients for F508del, Castaldo et al. found that 3 years of lumacaftor/ivacaftor significantly improved liver function parameters, including total and conjugated bilirubin, while the subsequent ETI treatment caused a significant increase in these parameters [25]. Recent investigations demonstrated that prolonged exposure to tezacaftor—a key component in ETI—acts as a direct inhibitor of the sphingolipid delta-4 desaturase enzyme (DEGS). This inhibition leads to the accumulation of dihydroceramides (dHCer) in human epithelial cells, hepatocytes, and brain tissue in vitro [26]. Concerns about liver toxicity have also been raised by the association between ETI and drug-induced liver injury reported by the FDA Adverse Event Reporting System (FAERS) [27]. Future studies are needed to establish the causal relationship between ETI and liver injury and the mechanisms of ETI-induced liver disease.

Given that the increase in ALT and conjugated bilirubin was temporary, although recurrent, close monitoring of liver biochemistry is recommended until laboratory values return to normal. Although the role of ursodeoxycholic acid in liver disease is debated, there was a significant increase in the number of pwCF taking this drug after the start of ETI. In pwCF with recurrent increases in liver enzymes, dose adjustments may be considered as a management option.

### 3.2. Effectiveness

The beneficial effects of ETI on lung function, antibiotic therapy cycles, SwCl, nutritional status, and daily respiratory symptoms were achieved and sustained throughout the two years of treatment. FEV_1_ is a strong predictor of clinical status in CF, with its decline closely linked to survival [28,29]. In our cohort of patients with severe lung disease, the overall average improvement in ppFEV_1_ over 24 months was 11.8 percentage points. This finding is consistent with those from a larger French study and corroborates a recent Italian single-center study, both of which reported sustained improvements in lung function over two years of follow-up [18,20]. Unlike the French study and the phase 3 trial, our data showed a rapid initial increase in FEV_1_ within the first weeks of treatment, followed by a slower improvement in pulmonary function. This finding likely reflects the real-life clinical variability in pwCF with severe pulmonary disease, who initiated ETI during either stable or unstable periods.

Previous studies have identified three exacerbations per year as a critical threshold for lung function decline in CF, with pwCF experiencing more than two exacerbations/year at increased risk of death or lung transplantation within three years [30]. Our study showed that ETI had a sustained impact on both pulmonary function and the need for antibiotic therapy, suggesting potential disease-modifying effects that could improve life expectancy in patients with severe CF. Notably, half of the patients already on the lung transplant list were delisted, and we observed a reduction in the number of patients requiring long-term oxygen therapy and non-invasive ventilation, which is consistent with findings from other studies [15,16,17,20].

In line with the literature, we observed a mean reduction in SwCl of −43.7 (−47.6, −39.9) mmol/L, over the two 2-year periods, reflecting a significant improvement in CFTR function. However, the correlation between the changes in SwCl and ppFEV_1_ was weak, with a concordance index (Cohen’s K) of 0.247. Similar to previous studies on CFTR triple-modulator therapy, our findings emphasize the need to further investigate the variability of sweat chloride levels and their relationship to clinical outcomes [31,32]. Moreover, early and persistent structural lung damage and residual infection burden in severe lung disease could contribute to a slight change in ppFEV1 during ETI treatment.

As shown in Table 4, the use of daily chronic medications decreased during ETI treatment. A recent single-center study of pwCF with mild-to-moderate lung disease on ETI therapy demonstrated a non-inferior lung function improvement in patients who reduced supportive therapies compared to their own controls [33]. While we did not assess whether reductions in supportive therapies were physician-approved, we found that clinical and functional benefits were maintained despite a decrease in medication use. Further prospective case–control studies are needed to better explore the possibility of safe reduction in supportive treatment in severe pwCF in the era of CFTR modulators.

Regarding our microbiological findings, ETI therapy was associated with minimal changes in sputum culture results, as PA was eradicated in only 3/82 (3.7%) patients during ETI treatment. This finding is not unexpected given the chronicity of PA infection and the severity of lung disease in our cohort.

One of the advantages of the present study is that it represents a real-life investigation on the benefits of ETI in CF patients with advanced lung disease outside the strictly defined criteria of a randomized controlled trial and presents noteworthy real-world applicability in everyday clinical practice.

On the other hand, our study has several limitations.

This was an observational multicenter study with retrospective data collection, so data are not homogenously collected. Because of the coronavirus disease (COVID-19) pandemic, some outpatient visits were skipped, and consequently, clinical data were missing for some centers. The COVID-19 pandemic may have contributed to the decrease in the number of PExs and antibiotic therapy cycles, due to limited social interaction and increased isolation during lockdowns. Additionally, our sample size limited subgroup analyses, particularly in pwCF with comorbidities such as CF-related liver disease. The observational nature of the study design limits the proof of a causal relationship between our clinical findings and ETI treatment.

Finally, the two-year follow-up period may have been insufficient to fully capture the long-term benefits and safety of ETI, and extending observation to 4–5 years may provide more comprehensive insights.

In conclusion, in CF patients with advanced lung disease on ETI treatment, we observed improvement in a number of clinically significant outcomes over a 2-year study period. However, several additional observations, such as liver dysfunction, variable degrees of lung function improvement, and limited impact on chronic airway infection, underscore the fact that the benefit–risk profile of ETI treatment in cystic fibrosis patients with advanced lung disease has not been fully elucidated and warrants prolonged-term monitoring.

## 4. Materials and Methods

### 4.1. Cohort and Study Design

A total of 18/27 (66.7%) Italian CF centers enrolled in an ETI compassionate use program participated in this cohort study with retrospective data collection [34]. The program was run between October 2019 and May 2020 by Vertex Pharmaceuticals with the purpose of providing ETI to pwCF heterozygous for F508del and with an MF mutation with severe disease, free of charge, whilst awaiting national funding approval. The pwCF continued the modulator therapy after the national reimbursement of commercialization of ETI started on 7 July 2021.

Demographic and clinical data were collected from patient files for 2 years prior to and 2 years after ETI was initiated, that is, time 0 (T_0_). All follow-up visits, both as out- and in-patients, were considered. Because of the coronavirus disease (COVID-19) pandemic, the exact timing of the visits was left at the referral physician’s discretion. Clinical care and chronic medications of participating patients were managed by the CF centers’ physicians.

The study was submitted and approved by the Ethical Committee of all Centers, and approval for the coordinating center was obtained on the 8 September 2021 (protocol number 46629). Written informed consent was obtained from all patients before data collection.

Data extraction occurred between March and June 2024. Vertex Pharmaceuticals played no role in the acquisition or analysis of data, in the writing of the manuscript, or in the decision to submit this manuscript.

### 4.2. Study Subjects

Criteria for entering the compassionate use program were as follows: (1) age 12 years or older; (2) heterozygosity for F508del and a MF mutation; and (3) advanced respiratory disease, defined as either highest ppFEV_1_ < 40% in at least the preceding 2 months, or being on a lung transplant waiting list or under assessment for listing [35].

Exclusion criteria included mechanical ventilation, severe hepatic impairment (class C of Child–Pugh score), history of advanced liver disease with or without hepatic impairment, history of solid organ or hematological transplantation, known history of alcohol or drug abuse in the past year, pregnancy, and sexually active patients of reproductive potential who were not willing to use appropriate contraception methods.

Treatment was administered orally according to the manufacturer’s recommendations (200 mg elexacaftor/100 mg tezacaftor/150 mg ivacaftor in the morning and 150 mg ivacaftor in the evening).

### 4.3. Outcome Measures

Safety was considered the primary outcome measure and was assessed through the treatment-related adverse events reported in patient charts by the CF center physicians. Moreover, the number of pwCF who discontinued and interrupted ETI treatment and who had an increase in levels of alanine and aspartate aminotransferase, y-glutamyl transferase, conjugated and total bilirubin, and creatine kinase greater than either two or three times the upper limit of the normal range (ULN) were reported.

The secondary outcome measure, planned to assess the effectiveness, was the difference between pre- and post-ETI treatment of the following parameters: ppFEV_1_, percent-predicted forced vital capacity (ppFVC), FEV_1_/FVC, the ratio between oxygen saturation of hemoglobin measured by pulse oximetry and the fraction of inspired oxygen (SpO_2_/FiO_2_), when long-term oxygen therapy was prescribed, sweat chloride (SwCl), the body mass index (BMI) z-score, the distance covered during the 6 min walking test (6 mWD), and the respiratory domain score of the Cystic Fibrosis Questionnaire-Revised (CFQ-R RD). Moreover, we assessed the absolute mean difference in the cumulative values recorded before and after the start of ETI of the following end points: annualized number of days of oral and intravenous (iv) antibiotic therapy and of hospitalization, and the annualized number of oral and iv antibiotic cycles and hospitalizations.

As a measure of effectiveness, we also evaluated the rate of change of ppFEV_1_ and BMI z-score during two different periods. To evaluate the early effect of therapy, we considered the last 6 months without modulator therapy and the first 6 months with ETI therapy. To evaluate the long-term effect of ETI, we considered the last 18 months following the start of ETI.

We were also interested in assessing the trend of chronic infection or prevalence of *Pseudomonas aeruginosa* positive cultures in the 2-year periods. As regards chronic medications, we compared the prevalence of pwCF treated with inhaled antibiotics, inhaled hypertonic saline, inhaled Dornase alfa, azithromycin, ursodeoxycholic acid, long-term oxygen therapy, and noninvasive ventilation in the pre-ETI and ETI periods.

### 4.4. Clinical Variables

Spirometry was performed according to the American Thoracic Society and European Respiratory Society criteria, and values were expressed as a percentage of predicted values using the Global Lung Function Initiative reference equations [36,37].

When long-term oxygen was delivered using nasal prongs or a Venturi mask system, the fraction of inspired oxygen (FiO_2_) was calculated according to the total flow: the FiO_2_ in room air was 0.21 [38]. The SpO_2_/FiO_2_ ratio allowed comparisons between patients breathing room air and those on different oxygen mixtures. SpO_2_ was measured in the sitting position before spirometry was undertaken. The 6 min walking test was performed according to the ATS standards [39].

BMI (weight in kilograms divided by the square of the height)-z score was calculated according to the CDC Growth Charts [40]. Respiratory symptoms were evaluated using the Cystic Fibrosis Questionnaire-Revised (CFQ-R): the respiratory domain (RD) score ranged from 0 to 100, with higher scores indicating a higher reported quality of life with regard to respiratory symptoms [41].

Chronic medications were recorded annually and evaluated across both 2-year periods. Chronic infection by *Pseudomonas aeruginosa* (PA) was defined if >50% of at least three sputum or pharyngeal swab cultures over each 2-year period were positive. The prevalence of PA was also assessed based on all available bacterial cultures before and after treatment initiation.

Mild or moderate liver disease was classified based on occasional abnormalities in liver function enzyme levels, hepatomegaly, and/or ultrasonographic evidence of hepatic steatosis for mild cases, or laboratory and ultrasonographic signs of portal hypertension for moderate cases.

### 4.5. Bias

All pwCF who met the inclusion criteria were considered, minimizing the risk of selection bias. No issues were reported in the administration of therapy, ruling out performance bias. There were no concerns related to diagnostic methods or outcome measurements (detection bias). Loss to follow-up was minimal and did not significantly affect the results (attrition bias). No other potential sources of systematic bias were identified.

### 4.6. Study Size

This is an observational study with retrospective data collection, including all eligible patients of 18 CF centers who were enrolled in the compassionate use program, as no formal sample size calculation was performed. The objectives were descriptive and exploratory, and the statistical analysis included an interrupted time series (ITS) model, which does not typically require a formal size estimation. However, with approximately 100 subjects, we expected to have more than 95% power to detect adverse events (main aim) with a frequency of around 3%.

### 4.7. Statistical Analysis

For each clinical variable, we considered the mean values recorded during each 2-year period, which preceded and followed the start of ETI.

A descriptive analysis of the main demographic and clinical variables was conducted, presenting the proportions for the qualitative variables and the median and quartiles (IQR) for the quantitative ones.

Treatment success was evaluated using a linear mixed model, including treatment period (pre-ETI vs. ETI) as a fixed effect and a random intercept for subjects.

The cumulative percentage of subjects achieving an increase of +10 or +5 percentage points in ppFEV_1_ after starting treatment over the two years of therapy was calculated. To express the pre- and post-treatment difference in the proportion of subjects taking various daily chronic medications, the McNemar test was used. To study the concordance between the pre- and post-treatment variation of ppFEV_1_ values and those of sweat chloride, we used Cohen’s K.

The comparison of ppFEV_1_ and BMI z-score pre- and post-therapy was also assessed using the interrupted time series analysis (ITS) in the period of one year before and after the beginning of therapy. For each subject, the same number of visits before and after therapy were selected, so that her/his values had the same weight in the two periods. Furthermore, the effect of therapy on ppFEV_1_ and BMI z-score at 6 months and 2 years was analyzed using paired Student’s *t*-test at the beginning of therapy (T_0_), the visit at 6 (±2) months (T_1_), and at 22 (±2) months (T_2_).

All statistical tests were two-tailed.

## Figures and Tables

**Table 1:** Participant demographic and clinical characteristics at baseline (T_0_).

Patient, no.	124
Sex, males no. (%)	64 (51.6)
Age, years, median (IQR)	34 (26, 43)
12–17 years, no. (%)	7 (5.6)
Adults, no. (%)	117 (94.4)
ppFEV_1_, percentage points, median (IQR)	34 (29, 41)
ppFEV_1_ < 35 percentage points, no. (%)	35 (28.2)
BMI z-score, median (IQR)	−0.83 (−1.63, −0.25)
Inclusion in waiting list for LT, no. (%)	16 (12.9)
Long-term oxygen therapy, no. (%)	44/114 (38.6)
Long-term noninvasive ventilation, no. (%)	16/115 (13.9)
Liver disease	
Mild °, no. (%)	40/112 (35.7)
Moderate °°, no. (%)	9/112 (8.0)
Diabetes ^#^, no. (%)	39/115 (33.9)

Values are median (interquartile range—IQR) or proportion of patients as a percentage. ppFEV_1_: percent-predicted forced expiratory volume in 1 s; BMI: body mass index; LT: lung transplantation; °: occasional abnormalities in liver function enzymes, hepatomegaly, and ultrasonographic evidence of hepatic steatosis; °°: laboratory and ultrasonographic signs of portal hypertension; ^#^: pwCF with daily insulin therapy.

**Table 2:** Drug-related adverse events.

Adverse Event	Number of Patients (Percent)
Rash	9 (7.3)
Acne	5 (4.0)
Rhinitis	1 (0.8)
Change in appearance	4 (3.2)
Dyspnea	4 (3.2)
Hemoptysis	2 (1.6)
Nausea	2 (1.6)
Diarrhea	2 (1.6)
Intestinal meteorism	5 (4.0)
Abdominal pain	4 (3.2)
Constipation	4 (3.2)
Headache	2 (1.6)
Joint pain	4 (3.2)
Muscle pain at the upper and lower extremities	2 (1.6)
Anxiety and depression	1 (0.8)

**Table 3:** Mean of the patient averages during the pre-ETI and ETI treatment periods and estimated mean differences between periods (ETI–pre-ETI) with 95% confidence intervals. Estimates were obtained from a linear mixed model with random subject intercept and treatment period as fixed effect.

Variables	PRE-ETI Period	ETI Period	Mean Difference	95% CI of Mean Diff	*p* Value
ppFEV_1_ (percentage points), mean	38.2	51.0	11.8	11.1 to 12.6	<0.001
ppFVC (percentage points), mean	57.5	71.4	13.3	12.4 to 14.1	<0.001
FEV_1_/FVC, mean	0.560	0.595	0.026	0.020 to 0.033	<0.001
SpO_2_/FiO_2_, mean	450.3	461.9	11.2	8.7 to 13.7	<0.001
6 MWD (meters), mean	557.5	608.6	52.4	39.3 to 65.5	<0.001
BMI z-score (standard deviation), mean	−0.93	−0.26	0.65	0.61 to 0.69	<0.001
CFQ-R RD (points), mean	65.4	87.8	23.2	20.4 to 26.2	<0.001
Sweat chloride (mmol/L), mean	100.8	57.5	−43.7	−47.6 to −39.9	<0.001
Oral antibiotic, courses/year	3.6	1.2	−2.2	−2.5 to −1.9	<0.001
Oral antibiotic, days/year	51.9	22.0	−26.7	−32.4 to −21.0	<0.001
Iv antibiotic, courses/year	2.4	0.6	−1.8	−2.0 to −1.5	<0.001
Iv antibiotic, days/year	34.1	10.5	−23.7	−27.6 to −19.9	<0.001
Hospitalization, number/year	2.1	0.5	−1.4	−1.6 to −1.2	<0.001
Hospitalization, days/year	28.7	6.5	−21.1	−24.2 to −18.1	<0.001

ppFEV_1_: percentage of predicted forced expiratory volume in one second; ppFVC: percentage of predicted forced vital capacity; SpO_2_/FiO_2_: ratio between oxygen saturation of hemoglobin measured by pulse oximetry and the fraction of inspired oxygen; 6 MWD: walking distance recorded during the 6 min walking test; BMI: body mass index; CFQ-R RD: the respiratory domain of the Cystic Fibrosis Questionnaire-Revised; Iv: intravenous.

**Table 4:** Use of chronic daily medications for the pre-ETI and ETI treatment periods.

Medication	N	Pre-ETI Period	ETI-Period	*p* Value
Pancreatic enzymes, no. (%)	115	114 (99.1%)	113 (98.3%)	0.317
Ursodeoxycholic acid, no. (%)	115	63 (54.8%)	71 (61.70%)	0.005
Azithromycin, no. (%)	115	62 (53.9%)	33 (28.7%)	<0.001
Insulin, no. (%)	115	39 (33.9%)	35 (30.4%)	0.045
Inhaled antibiotics, no. (%)	109	87 (79.8%)	72 (66.1%)	0.001
Inhaled Dornase alfa, no. (%)	115	76 (66.1%)	62 (53.9%)	0.001
Inhaled hypertonic saline, no. (%)	115	64 (55.7%)	57 (49.6%)	0.090
Long-term oxygen therapy, no. (%)	114	44 (38.6%)	19 (16.7%)	<0.001
Long-term noninvasive ventilation, no. (%)	115	16 (13.9%)	6 (5.2%)	0.002

## Data Availability

The original contributions presented in this study are included in the article and Supplementary Materials. Further inquiries can be directed to the corresponding author.

## Supplementary Materials

The following supporting information can be downloaded at https://www.mdpi.com/article/10.3390/ijms262110513/s1.

## Appendix A

Principal investigators and study coordinators of the Italian SITTMA Study Group:

Esposito Irene ^1^, Laura Barrocu ^1^, Barbara Messore ^2^, Sara Demichelis ^2^ (Turin), Carla Colombo ^1^, Arianna Biffi ^1^, Andrea Gramegna ^2^, Maura Spotti ^2^ (Milan); Sonia Volpi, Ilaria Meneghelli (Verona); Ugo Pradal, Elena Proietti (Rovereto—Trento); Mirco Ros, Isabella Comello (Treviso); Federico Cresta, Maria Lombardi (Genova); Giovanna Pisi, Cinzia Spaggiari (Parma); Maura Ambroni, Valentina Donati (Cesena); Giuseppe Cimino, Maria Angela Ciminelli (Rome); Federico Alghisi, Fabiana Ciciriello (Rome); Pietro Ripani, Stefano Pantani (Atri); Pamella Vitullo, Mariaantonietta Ciciretti (Cerignola—Foggia); Giuseppina Leonetti, Domenica De Venuto (Bari); Donatello Salvatore, Angela Pepe (Potenza); Maria Cristina Lucanto, Maria Ausilia Catena (Messina); Francesca Ficili, Stefana Barrale (Palermo); Leonardi Salvatore, Giuseppe Fabio Parisi (Catania); and Daniela Manunza (Cagliari).

^1^  Paediatric Cystic Fibrosis Centre;

^2^  Adult Cystic Fibrosis Centre.
