# Supplementary material for: Real-World Safety and Effectiveness of Elexacaftor, Tezacaftor, and Ivacaftor in People with Cystic Fibrosis and Advanced Lung Disease: A Two-Year Multicenter Cohort Study
Source: Int J Mol Sci. 2025 Oct 29;26(21):10513. doi: 10.3390/ijms262110513 (PMC12609796; doi:10.3390/ijms262110513)

Supplementary Figure S1. Individual differences of 123 subjects with respect to percentage of predicted FEV_1 between the mean values of pre-ETI and ETI treatment periods. The dashed line indicates the mean value.

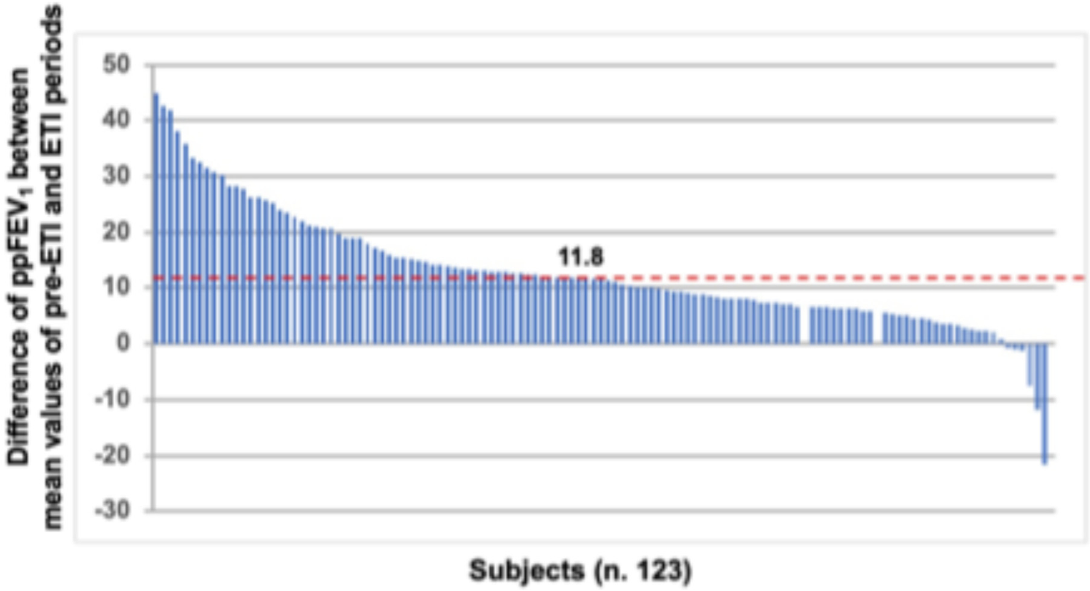

Supplementary Figure S2. Cumulative percentage of subjects who achieve an increase in percent of predicted FEV_1 by at least 5 (blue line) or 10 (red line) percentage points after the start of treatment (T_0)

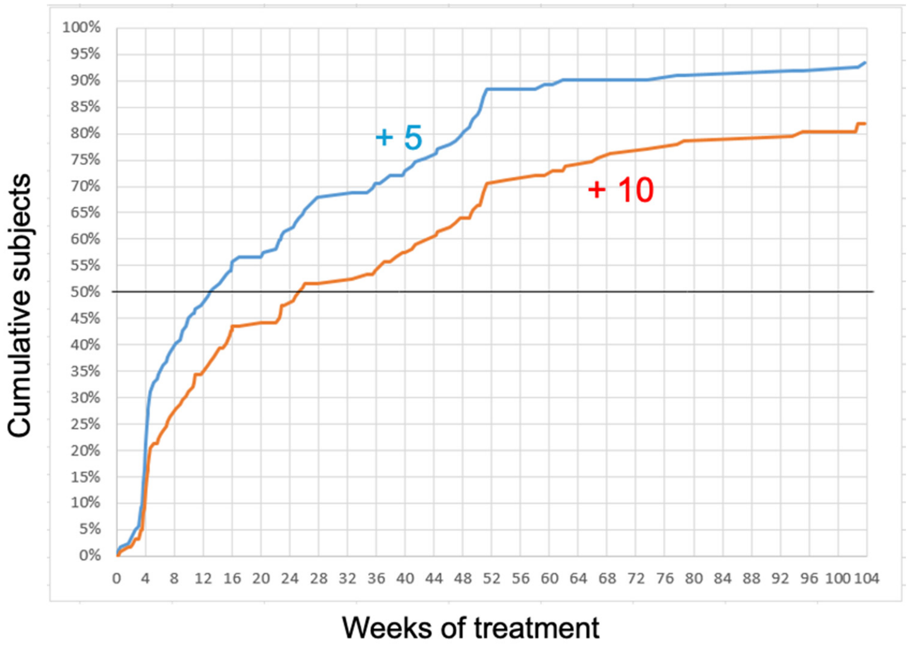

Supplementary Figure S3. Relationship between the percentage of predicted FEV_1 ($ppFEV_1$) and values of sweat chloride (SwCl) in 94 subjects. For both variables the mean individual differences between the mean values of the pre-ETI and ETI treatment periods were shown. The red lines indicate the difference of 5 percentage points and 20 mmol/L in $ppFEV_1$ and SwCl, respectively.

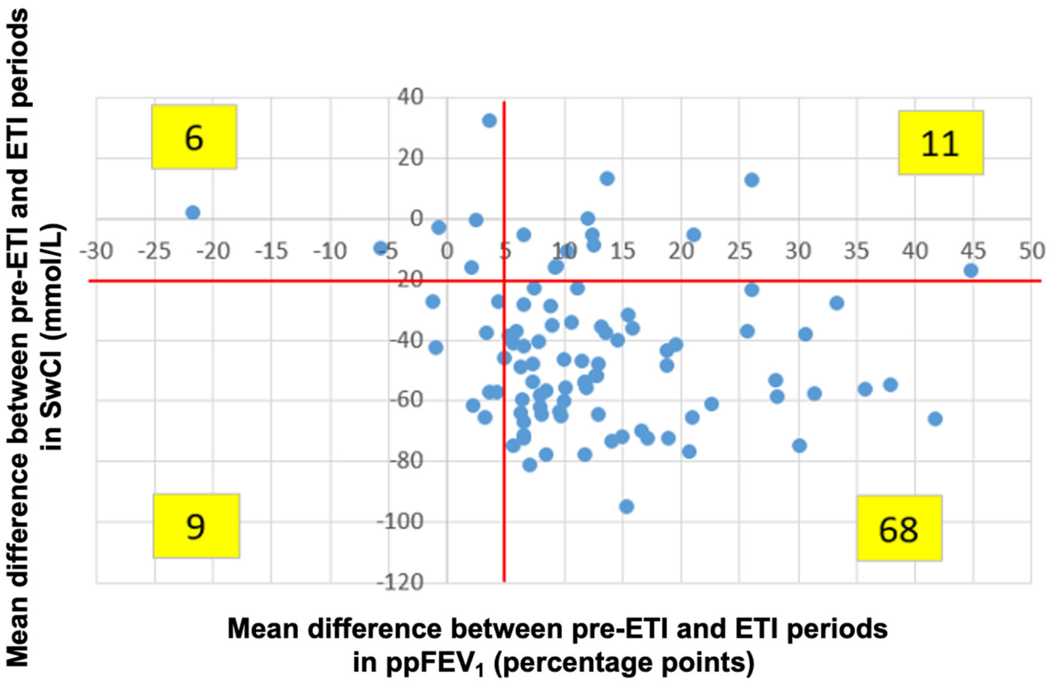

Supplement: Supplementary file 1 [file ijms-26-10513-s001.zip › ijms-3949426-supplementary.pdf]
